# Single click automated breast planning with iterative optimization

**DOI:** 10.1002/acm2.13033

**Published:** 2020-10-05

**Authors:** Ben Archibald‐Heeren, Mikel Byrne, Yunfei Hu, Guilin Liu, Nick Collett, Meng Cai, Yang Wang

**Affiliations:** ^1^ Icon Cancer Centres Wahroonga NSW Australia

**Keywords:** automation, automated planning, radiotherapy planning, breast radiotherapy, hybrid IMRT

## Abstract

**Purpose:**

To present the development of an in‐house coded solution for treatment planning of tangential breast radiotherapy that creates single click plans by emulating the iterative optimization process of human dosimetrists.

**Method:**

One hundred clinical breast cancer patients were retrospectively planned with an automated planning (AP) code incorporating the hybrid intensity‐modulated radiotherapy (IMRT) approach. The code automates all planning processes including plan generation, beam generation, gantry and collimator angle determination, open segments and dynamic IMRT fluence and calculations. Thirty‐nine dose volume histogram (DVH) metrics taken from three international recommendations were compared between the automated and clinical plans (CP), along with median interquartile analysis of the DVH distributions. Total planning time and delivery QA were also compared between the plan sets.

**Results:**

Of the 39 planning metrics analyzed 23 showed no significant difference between clinical and automated planning techniques. Of the 16 metrics with statistically significant variations, 2 were improved in the clinical plans in comparison to 14 improved in the AP plans. Automated plans produced a greater number of ideal plans against international guidelines as per EviQ (AP:77%, CP:68%), RTOG 1005 (AP:80%, CP:71%), and London Cancer references (AP:80%, CP:75%). Delivery QA results for both techniques were equivalent. Automated planning techniques resulted in an average reduction in planning time from 23 to 5 minutes.

**Conclusion:**

We have introduced an automated planning code with iterative optimization that produces equivalent quality plans to manual clinical planning. The resultant change in workflow results in a reduction in treatment planning times.

## 1 INTRODUCTION

Breast cancer is the most common cancer in women worldwide, with almost 1.7 million diagnoses in 2012 or 25% of all cancer diagnoses in women.^1^ Incidence rates tend to be significantly higher in countries of higher levels of development with rates per 100,000 populations of 111.9, 95.0, 92.9, and 86.0 for Denmark, United Kingdom, USA, and Australia, respectively.^2^ Radiotherapy has a significant role to play in the treatment of breast cancer; of the 16,000 incidences of breast cancer in Australia in 2015, a total of 13,969 (approximately 87%) cases received radiotherapy treatment.^3^

With such a large number of clinical cases, breast radiotherapy shows the potential for significant efficiency gains with the introduction of automation. This work focuses on the potential reduction in planning times and changes in the plan‐approval workflow in breast radiotherapy.

Previous papers in the literature have described methodologies for automated planning of breast patients using heuristic optimization,^4^, ^5^ external markers,^4^, ^5^, ^6^ database libraries,^7^, ^8^, ^9^ semiautomatic optimization by component protocol scripts,^9^, ^10^ and automatic optimization following manual beam setup.^11^. Some initial papers are beginning to show promising results for machine learning‐based techniques for automated planning of breast patients.^12^ We instead propose a fully automated method (auto‐plan) that mimics the process adopted by human dosimetrists in iteratively optimizing with changing optimization weights dependent on plan performance against clinical objectives. To the author’s knowledge this is the first presentation in the literature of a fully automated methodology that does not require a database of reference plans, nor hard‐coded heuristic optimization objectives; instead, planning objectives are optimized iteratively in response to patient geometry/planning challenges.

The paper investigates the validity of such an approach for fully automated lumpectomy breast patient plans, including comparisons of plan time, consistency, quality, and deliverability with those of manually created plans that were previously accepted clinically.

## 2 MATERIALS AND METHOD

### 2.1 Patient cohort

All comparisons in this paper were performed retrospectively on a cohort of clinical patients planned with tangential hybrid breast radiotherapy at the site between June 2016 and November 2017. Patients were excluded from the study where nodal irradiation was included, or where a mastectomy had been performed. A total of 100 patients were selected for the study. All patients within the cohort were of stage 0, I, II breast ductal carcinoma in situ. All patient data used in this study were anonymized and all analysis and data mining were performed under the approval of the local institutional ethics board.

At the authors department two dose and fraction regimes are permitted for breast radiotherapy, namely 5000cGy in 25 fractions and 4240cGy in 16 fractions. The distribution of patients in this study was 57 right breast (40 at 5000cGy, 17 at 4240cGy) and 43 left breast (32 at 5000cGy, 11 at 4240cGy) patients. All patients within the study had received and completed whole breast hybrid IMRT tangent radiotherapy.

### 2.2 Clinical planning

The hybrid technique has been shown to provide high‐quality plans that are robust to patient breathing motion.^13^, ^14^, ^15^, ^16^ The primary characteristic of the technique is conformal fields that deliver the majority of breast dose accompanied with a small weighting of modulated multileaf collimator (MLC) fields to fine tune the distribution.

Clinical plans (CPs) in this study applied the hybrid technique using an extensive clinical protocol with asymmetric conformal tangential fields and dynamic MLC (DMLC) modulated tuning fields. Plan parameters including jaw size, beam energy, MLC distribution, beam weighting, gantry angle, collimator, angle and isocenter placement were all determined by the dosimetrists to achieve the best possible dose distribution. Evaluation of plan performance was aided in clinical practice by conformance to a list of clinical DVH criteria (clinical goals). Open fields were weighted to give approximately 85% of the total target dose, with maximum dose from open fields limited to 95% of the total target dose. IMRT fields of identical geometries were then added to improve the plan by ensuring optimal coverage with minimal hotspots. To prevent fluence escalation at the skin from surface optimization,^13^, ^17^ the PTV was retracted 5mm from the skin for planning. Target coverage with setup variations was ensured by adding “flash,” where MLCs are extended past the typical PTV margin to account for breathing motion, to the open beams. By delivering the prescribed dose predominantly by the open beams, there was no need for anterior MLC “flash” in the IMRT beams.

### 2.3 Automated Planning

The automated plans (APs) were created using code written in IronPython 2.7 and run through the Raystation 6.0 planning system (Raysearch Laboratories, Stockholm, Sweden) scripting environment. The entire code implementation is the combination of several internally developed modules, which are created to run on the standard Raystation IMRT license without installing further python libraries and the need of previous plan databases.

**Figure 1:**
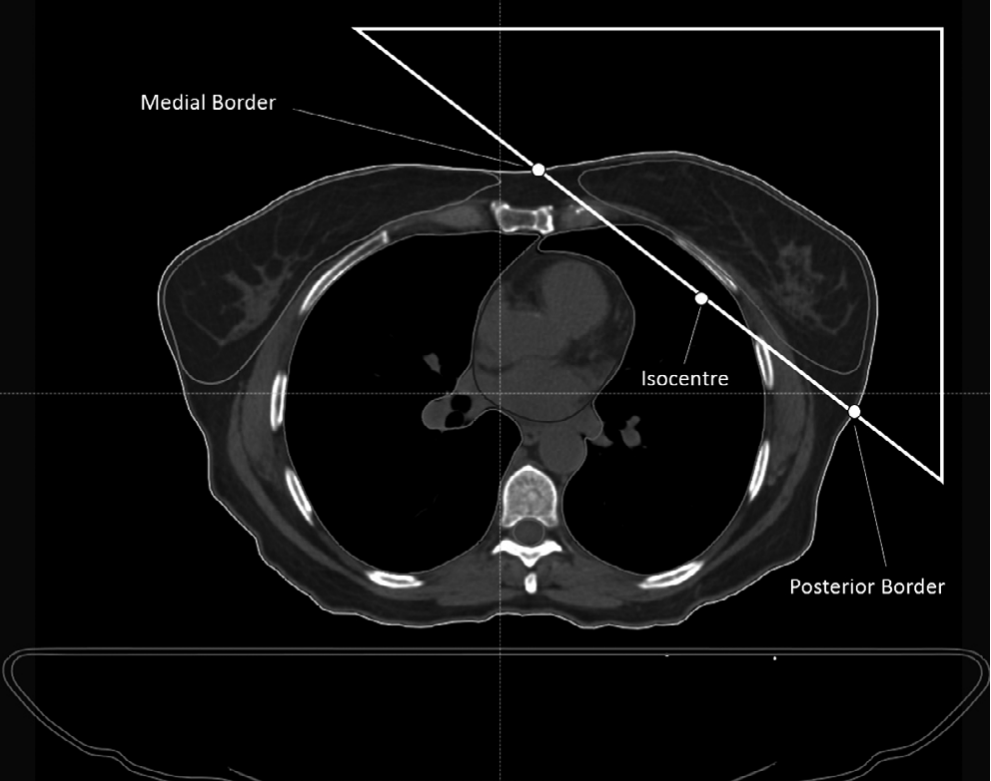
An adequately contoured dataset for iterative Breast auto‐planning (isocenter and borders are shown for clarity, they are created in the auto‐planning code and are not a requirement of the software).

The AP code is reliant on approved contours (Figure 1) from the oncologist/dosimetrist for standard breast planning. These are outlined in guidelines set out by ESTRO^18^ and RTOG^19^ and include;

‐ Whole Breast PTV
‐ Ipsilateral Lung
‐ Contralateral Lung
‐ Heart
‐ Contralateral Breast
‐ Liver
‐ Body/External
‐ Support Structures (Couch)

In some clinical cases a CTV boost volume is included clinically for a sequential boost. Plans for sequential boosts were not included in this study. The CTV for the whole breast PTV is the palpable breast.

These contours are generated by auto‐segmentation code not explored in this paper. The auto‐generation is a combination of atlas‐based segmentation, heuristic ROI algebra, and intensity‐based structure optimization. Also created automatically are relevant tattoo markers, a tangent geometry, medial and posterior border points, and planning structures (including rings and dose control volumes). All auto‐segmented structures are reviewed and edited as necessary by Oncologists prior to planning.

On initiation of the AP code, a user interface is presented. Users are required to specify target dose, fractionation, treatment machine, delivery technique, the primary PTV, and any boost PTV or nodal PTV to be treated. For the purpose of this paper the breast PTV was selected to be the only PTV and the Hybrid technique was selected as the only planning technique. All CPs and APs were created with a Varian iX beam model. The machine is dual energy (6MV and 18MV), with a millennium 120 leaf MLC of 0.5cm leaf width centrally (inner 80 leaves) and 1cm leaf width at larger field sizes (outer 40 leaves). The machine has a maximum possible field size of 40cm x 40cm with leaf modulation possible along all MLC pairs.

The automated planning functionality performed by this code includes;

### 2.4 Automated beam generation

The tangent beam geometry is created by the auto‐segmentation code. The position is optimized by incremental translation and affine transformations such that the intersection of the tangent with the external contours approaches the intersection of the tangent and PTV plus a volume margin to account for the field edge‐to‐PTV margin. The optimization of the tangent geometry is simplified by a condition of entry through the midline tattoo as per the protocol of the center which primarily limits the transformations to rotations pinned at the midline tattoo. An optimized tangent geometry is shown in Figure 1. The medial and posterior border are calculated from the intersection of the posterior aspect of the tangent with the tissue edge at the superior/inferior mid‐plane level of the PTV. This is performed through a series of code driven algebraic functions that determine the outer rim of the tangent geometry and external contours and take their intersections at the level of the midline tattoo.

Simple trigonometry (equations 1 and 2) is utilized to determine the optimal medial gantry angle for the tangent beams, rounded to the nearest full angle as per clinical protocols at the site. Lateral angles are taken at the opposing angle to promote a closed‐jaw nondivergent posterior edge.

For left‐sided breast patients;

(1) $$\theta_{m}=360‐\tan^{‐1}\left(\frac{x_{m}‐x_{p}}{y_{m}‐y_{p}}\right)$$

For right‐sided breast patients;

(2) $$\theta_{m}=\tan^{‐1}\left(\frac{x_{m}‐x_{p}}{y_{m}‐y_{p}}\right)$$

where;

- (*x_m_*, *y_m_*) are the x and y (lateral and anterior–posterior) DICOM coordinates of the medial border,
- (*x_p_*, *y_p_*) are the DICOM coordinates of the posterior border; and
- θ_m_ is the medial and posterior tangent gantry angles.

Isocenter placement (figure 1) is determined by the mid separation of the posterior beam edge (equation 3). The isocenter position allows for a nondivergent posterior edge (i.e., posterior jaw = 0.0cm).

(3) $$x_{\mathrm{iso}},y_{\mathrm{iso}}=\left\{\begin{array}{cc} \frac{x_{m}+x_{p}}{2},\frac{y_{m}+y_{p}}{2}, & \left|x_{m}‐x_{p}\right|<10,\\ x_{m}\pm 10),y_{m}\pm 10\times \left(\frac{y_{m}‐y_{p}}{x_{m}‐x_{p}}\right), & \left|x_{m}‐x_{p}\right|\geq 10 \end{array}\right)$$

where;

- *x_iso_*, *y_iso_* are the x and y co‐ordinates of the plan isocenter
- ± is dependent on laterality of patient

The isocenter placement is rounded along the tangent angle to ensure full centimeter shifts from the midline tattoo to facilitate setup. Jaw size, collimator angle, and MLC apertures are determined through Raystation 3D conformal radiotherapy beam optimization, to ensure that the mean dose from open segments accounts for approximately 85% of the prescribed dose, with equivalent weighting between medial and lateral beams. The optimization includes dose controls on heart and lung doses, minimum posterior PTV coverage, and maximum PTV dose to promote apertures that effectively spare the proximal organs at risk.

For very large patients, the code attempts mixed photon energy beams to improve target coverage. Where the straight‐line distance between the medial and posterior border (i.e., medial and posterior beam entry points) exceeds 25cm, the two open conformal fields are copied, and the resulting four beams are split equally between 6MV and 18MV. This process is implemented on suitable patients in clinical plans on discretion of the planner. 18MV fields were used in 6 of the 100 patients treated. Figure 1 provides a visual representation of the beam angle, medial border, and isocenter placement.

A secondary dynamic MLC intensity‐modulated radiotherapy (IMRT) beam set is created with beams of identical tangent collimator and gantry angles. The beam set is created such that objective functions are optimized with consideration of the initial open beam dose as background dose as per instructions from the well‐published hybrid IMRT technique.^14^, ^15^, ^16^, ^20^, ^21^ At this point the plan is passed to the iterative optimization code.

### 2.5 Iterative optimizer

The iterative optimizer is code developed by the authors within Raystation’s IronPython scripting environment and leverages the Raystation dose optimization algorithm. The code optimizes modulated radiotherapy deliveries by incremental changes to objective functions in response to changing plan quality. Changes in plan quality are periodically monitored by querying pass or failures of defined clinical goals. In this study the code is utilized for tangential breast radiotherapy. For various sites both objective functions and clinical goals are loaded from comma separated variable (CSV) reference files. Each objective function is linked by a search code to a clinical goal (CG) by reference of structure, structure objective/CG type, proximity of objective/CG dose, and dose volume histogram (DVH) metric. As an example, if a clinical goal for the left lung of V30Gy <10% existed, the code would search through the objectives for function for the left lung with dose objectives below 30Gy. In finding several functions it would link only to the closest dose level DVH metric objective function. In the cases of minimum dose goals, the search is limited to corresponding functions with higher target doses. Optimization is performed for 100 iterations before a full collapsed cone convolution (CCC) dose calculation. DVH statistics of the calculated dose are compared against clinical goals. Failing clinical goals result in an increased weighting for corresponding objective functions. The initial optimization is intentionally weighted to OAR sparing. This promotes simpler optimization problems where primarily target coverage and a competing OAR are the focus of optimization, rather than several simultaneous competing OARs.

In some favorable geometries clinical goals may all be easily met with minimal optimization. To prevent under‐planning, where dose reduction to organs is not as low as possible due to adherence of clinical goals in first instance calculations, any organ at risk objective function easily met (objective function value = 0) has its objective dose reduced to 90% of the achieved dose after CCC calculation and a new optimization loop will be initiated.

If any objective function, for either targets or OARS, requires adjustment, the loop is repeated from the 100 iterations of optimization with the newly weighted objective functions. As an example, let us say that the clinical goal for PTV coverage is 4750cGy to 95% and there is an objective linked to that clinical goal with a weighting of 100. Based on the proximity of the achieved coverage to the clinical goal, the objective function will be increased by a multiple between 1.2 and 3. As an example we set an appropriate factor as 2. The weighting is increased to 200 and the optimization runs for another round of iterations. The same would be true for a lung objective at a higher dose than its clinical target. This process is applied for all failing clinical goals at each iteration round.

The use of 100 iterations was chosen from testing on a separate cohort of 15 patients to determine the optimal parameters to achieve plan improving changes in a short amount of time but preventing narrow optimization that overly preferences a single particular failing goal.

**Figure 2:**
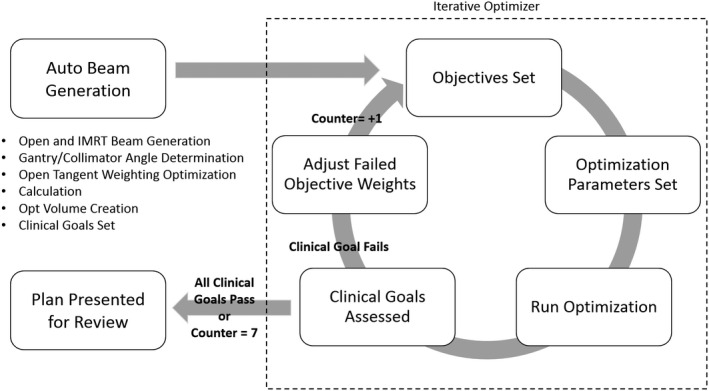
The iterative optimization workflow.

The code loops repeatedly until all clinical goals are met or until a predetermined maximum number of iterations (600) are reached. It is not expected that this maximum number of iterations will be reached, which typically reflects very difficult cases. In cases where all clinical goals are met this results in a single click planning process. In clinical practice, any failed dose goals after the maximum set iterations could be further optimized by the user. In this study, human changes after the iterative optimization were prohibited. A visual representation of the automated planning workflow is shown in Figure 2.

While other groups have provided heuristic‐ and knowledge‐based optimization,^4^, ^5^, ^6^, ^8^, ^9^ where optimal objective functions are predicted, the iterative nature of this code provides a method potentially more able to deal with large changes in patient geometry. Small patient changes that would result in poor plan quality with initial objective functions are tailored by the incremental adjustment of weightings, in the same manner that human dosimetrist would adjust IMRT weightings to improve plan quality. This also means that this iterative optimization code can be applied to any anatomical sites and delivery techniques without feeding it with additional rules or training sets, a potential advantage over machine learning or regression techniques.

All AP plans were created blind of the associated CPs, with no input of the quality of the retrospective CPs. All plans were free of any human interactions with the exception of starting the script. The automated plans were created on the identical protocol as the CPs and variations in beam geometry, weighting, and optimization are purely a result of variations between human and software interpretation of optimal solutions.

Following the iterative optimizer all plan DVH statistics were exported for analysis within the AP code.

### 2.6 Plan quality

Analysis was performed separately depending on the laterality of the disease (left or right). While two dose regimes exist and were used in planning, for simplicity of presentation all results were scaled to 5000cGy equivalent for evaluation. As an example, for a plan prescribed with the 4240 cGy dose regimen, a lung dose value of 424cGy will have the analysis DVH data scaled by 5000/4240, resulting in a value of 500cGy.

Evaluation of the plan was performed by DVH comparison and assessment of specific clinical targets against the original CPs. The volumes considered within the analysis were the PTV, ipsilateral lung, contralateral lung, combined lung, contralateral breast, and heart. The metrics assessed for the regions of interest included dose delivered to a certain volume (e.g., d50 = dose delivered to 50% of the volume), volume receiving a certain dose (V5 = percentage volume of structure receiving 500cGy), and mean dose (dMean). For left‐sided patients, three metrics were added for the heart that were unnecessary for the right‐sided patients. As such the total number of metrics analyzed were 18 for the right breast cases and 21 for the left. The distribution of each metric was checked to ensure it was normally distributed. For each evaluation metric, a paired two‐tail t test was performed to determine statistically significant variations in plan quality between the CPs and APs. Significance was determined at a *P* value < 0.05, with a *P* value < 0.005 considered highly significant.

A secondary DVH analysis was performed by comparison of plan quality against determined ideal and acceptable metrics (where provided) as outlined in three documents, the RTOG 1005 protocol,^22^ the Australian EviQ guidelines,^23^ and the London Cancer breast radiotherapy guidelines.^24^, ^25^ A summary of the constraints used for analysis are shown in Table 1.

**Table 1:** Ideal and Acceptable criteria per assessed protocols [16‐19]

Paper/Protocol	Target Constraints	Ideal Constraints	Acceptable Constraints
EviQ	PTV d95%> 95%	Ipsilateral Lung V20 (V16) < 15%	
		Heart V25 (V20) < 10%	
		Heart Mean<4Gy	
RTOG 1005	PTV d95%> 95%	Ipsilateral Lung V20 (V16)<15%	Ipsilateral Lung V20 (V16)<20%
	Max<110%	Ipsilateral Lung V10 (V8)<35%	Ipsilateral Lung V10 (V8)<40%
		Ipsilateral Lung V 5 (V4)<50%	Ipsilateral Lung V 5 (V4)<55%
		Contralateral Lung V5<10%	Contralateral Lung V5<15%
		Contralateral Breast<3.1Gy	Contralateral Breast<5Gy
		Contralateral Breast d5<1.9Gy	Contralateral Breast d5<3.1Gy
		Heart V10 (V8)<30%	Heart V10 (V8)<35%
		Heart V20 (V16)<5%	Heart V25 (V20)<5%
		Heart Mean<4Gy	Heart Mean<5Gy
London Cancer	PTV d95%> 95%	Ipsilateral Lung V22 (V18)<15%	
	Max<110%	Heart V15 (V13)<10%	
		Contralateral Lung V2.5<15%	
		Contralateral Lung Mean<2Gy	

A comparison of clinical and automated beam angles was performed across the 100‐patient cohort to assess the ability of the automated software to correctly identify the appropriate angles for a given patient geometry.

### 2.7 Deliverability

To ensure the created auto‐plans could be delivered on treatment, a random selection of 10 auto‐plans was delivered to an ArcCheck quality assurance (QA) device (Sun Nuclear Corporation, Florida, USA). Gamma analysis was performed at a global 3%/3mm criteria with a 10% threshold, the result of which was compared to that of the QA of the corresponding clinical plan.

### 2.8 Planning time

Of the 100 patients used in the plan quality study, a subset of 20 random patients was used to evaluate the potential efficiency gains of automated planning. In each of the 20 cases, an expert dosimetrist replanned the cases free from interruptions. Measurements were timed from the beginning of plan generation to the final calculation at the end of optimization when the dosimetrist deemed the plan fully optimized. To keep the measurements as similar as possible the same scripted timing code was incorporated for the manual and AP time measurements, and the AP plan dose was provided to the dosimetrist to prevent time costs of optimizing past the automated plan quality. As such this provides an approximate minimum time for a dosimetrist to recreate a known dose distribution using the software. Comparisons were made between the average planning time for the manual and automated plans for the 20 subset patients.

## 3 RESULTS

### 3.1 Plan quality

**Figure 3:**
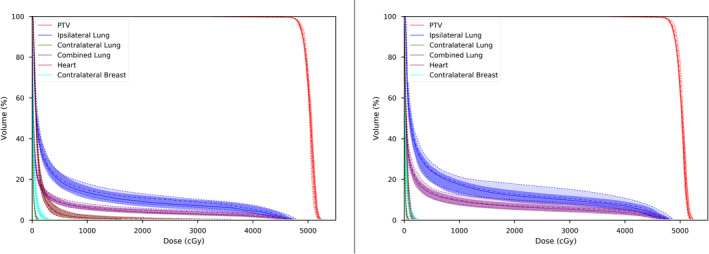
Median and first‐third quartile distributions for clinical (dotted lines) and automated (solid lines) plans for the (left) left and (right) right breast cases.

The dose volume histograms for both the CP and the AP plans of both left and right breast patients are shown in Figure 3. In each figure the median distributions of the automated and clinical plans are represented by the thin solid and dashed lines, respectively. The first and third quartiles of the distributions are represented by the opaque regions in the distributions.

Comparisons of the evaluation metrics for each dose regime and laterality are shown in Table 2. P values and significance in variance are displayed for each metric along with the dosimetrically superior planning technique.

**Table 2:** Statistical metric comparison between clinical manual plan and automated plan for right and left breast (all scaled to 5000cGy)

ROI	Metric	Lt Breast (N = 43)					Rt Breast (N = 57)				
		Clinical	Auto	Preferred Avg DVH	T Test p value	Statistical Significance	Clinical	Auto	Preferred Avg DVH	T Test *P* value	Statistical Significance
		**Avg (CI)**	**Avg (CI)**				**Avg (CI)**	**Avg (CI)**			
PTV_Breast	d98 (cGy)	4777 (4524‐5031)	4772 (4598‐4947)	Clinical	0.6291	Not Significant	4800 (4697‐4904)	4788 (4691‐4884)	Clinical	0.1004	Not Significant
	d95 (cGy)	4851 (4610‐5093)	4850 (4945‐4754)	Clinical	0.7969	Not Significant	4870 (4785‐4955)	4848 (4758‐4938)	**Clinical**	**0.0017**	**Highly Significant**
	dMean (cGy)	5011 (4772‐5249)	5032 (4970‐5095)	Auto	0.9221	Not Significant	5037 (4944‐5129)	5021 (4968‐5075)	**Auto**	**0.0161**	**Significant**
	d2 (cGy)	5161 (4923‐5399)	5192 (5092‐5293)	Clinical	0.0943	Not Significant	5194 (4941‐5448)	5178 (5075‐5280)	Auto	0.2961	Not Significant
Ipsilateral Lung	V5 (%)	21.2 (8‐34.2)	20.3 (8.8‐31.7)	Auto	0.0756	Not Significant	26.0 (12.2‐35.5)	22.4 (12.2‐32.6)	**Auto**	**0.0026**	**Highly Significant**
	V20 (%)	11.3 (1.4‐2121.1)	9.9 (1.7‐18.1)	**Auto**	**0.0001**	**Highly Significant**	11.9 (4.2‐22.1)	11.7 (3.9‐19.6)	**Auto**	**0.0001**	**Highly Significant**
	V30 (%)	9.1 (1.0‐17.3)	7.9 (0.7‐15.1)	**Auto**	**0.0001**	**Highly Significant**	10.6 (2.7‐19.4)	9.2 (2.3‐16.9)	**Auto**	**0.0001**	**Highly Significant**
	dMean (cGy)	609 (181‐1037)	554 (191‐916)	**Auto**	**0.0246**	**Significant**	722 (303‐1140)	652 (294‐1011)	**Auto**	**0.0001**	**Highly Significant**
	d2 (cGy)	4424 (3616‐5232)	4394 (3655‐5132)	Auto	0.0618	Not Significant	4596 (3924‐5268)	4549 (3926‐5174)	**Auto**	**0.0008**	**Highly Significant**
Contralateral Lung	dMean (cGy)	17 (7‐28)	18 (6‐29)	Clinical	0.1293	Not Significant	12 (1‐23)	11 (1‐22)	Auto	0.3349	Not Significant
	d5 (cGy)	48 (27‐69)	50 (20‐79)	Clinical	0.2496	Not Significant	36 (8‐65)	35 (9‐60)	Auto	0.3173	Not Significant
Combined Lung	dMean (cGy)	287 (82‐493)	261 (91‐431)	**Auto**	**0.0012**	**Highly Significant**	412 (165‐658)	371 (157‐586)	**Auto**	**0.0001**	**Highly Significant**
	d5 (cGy)	2065 (0‐4375)	1753 (0‐3875)	**Auto**	**0.0034**	**Highly Significant**	3298 (939‐5657)	2970 (526‐5415)	**Auto**	**0.0001**	**Highly Significant**
Heart	V25 (%)	1.1 (0.0‐4.9)	1.1 (0.0‐4.8)	None	0.8838	Not Significant	0	0	None	0.3223	Not Significant*
	V20 (%)	1.3 (0.0‐6.2)	1.2 (0.0‐5.5)	Auto	0.8109	Not Significant	0	0	None	0.3223	Not Significant*
	V10 (%)	2.2 (0.0‐8.7)	1.9 (0.0‐7.4)	Auto	0.7025	Not Significant	0	0	None	0.3071	Not Significant*
	dMean (cGy)	166 (0‐382)	153 (0‐342)	Auto	0.0512	Not Significant	44 (17‐71)	42 (15‐69)	Auto	0.1488	Not Significant
	d2 (cGy)	1209 (0‐3665)	1136 (0‐3244)	Auto	0.6806	Not Significant	149 (48‐250)	139 (36‐242)	**Auto**	**0.0198**	**Significant**
Contralateral Breast	dMean (cGy)	34.5 (9‐60)	36.5 (9‐64)	**Clinical**	**0.0355**	**Significant**	30 (5‐53)	28 (9‐48)	Auto	0.5074	Not Significant
	d5 (cGy)	122.6 (48‐197)	127.2 (40‐214)	Clinical	0.4041	Not Significant	113 (35‐191)	107 (50‐163)	Auto	0.2877	Not Significant
	d2 (cGy)	172.6 (64‐281)	187.3 (62‐313)	Clinical	0.0711	Not Significant	159 (62‐256)	157 (67‐246)	Auto	0.9744	Not Significant

CI = Confidence Interval taken as 1.96σ from mean.

a *Data not included in analysis.

Significance *P*< 0.05 (Bold).

Of a total of 39 metrics analyzed, 23 showed no significant variation between CPs and APs. Of the 16 metrics that did show statistically significant variations, 14 were improved on the APs.

The two metrics in which the CPs were superior were d95 PTV coverage in right breast patients, and the mean dose of the contralateral breast in left‐sided patients. The average improvements in these metrics were 22cGy and 2cGy, respectively.

In comparison against national/international breast protocols the AP plans adhered to 77%, 80%, and 80% of cases for ideal constraints of RTOG, EviQ, and London Cancer guidelines, respectively. Left‐sided cases had lower ideal plan rates (RTOG: 34/47, EviQ: 37/47, London Cancer: 36/47) than right‐sided cases (RTOG: 43/53, EviQ: 43/53, London Cancer: 44/53).

CPs had a consistently lower adherence to guidelines, with 68% of cases meeting constraints for RTOG guidelines, 71% for EviQ, and 75% for London Cancer dose recommendations. CPs demonstrated the same poorer plan performance for left‐sided cases in comparison to right‐sided cases.

Both the CPs and APs met all acceptable criteria of the RTOG guidelines in all cases.

Of the 100 patients analyzed, the variation in automated and planned beam angle was within 1 degree in 72% of cases (0°‐ 34, 1°‐ 38) with an overall mean variation of 0.17 +/‐1.97 degrees. For the 28 cases in which the gantry varied by more than 1 degree (2°‐ 8, 3°‐ 5, 4°‐ 11, 5°‐ 4) the auto‐segmentation code selected gantry angles that irradiated less tissue in 89% of cases (25/28).

### 3.2 Deliverability

For the 10 delivered patients the average percentage of points passing the gamma criteria were 97.9% (+/‐ 2.5%) and 99.4% (+/‐ 0.4%) for the clinical and AP deliveries, respectively. *P* = 0.0714, indicating no statistically significant difference.

### 3.3 Planning time

Table 3 shows the average, maximum, and minimum planning times for each of the cohorts between clinical and automatic produced plans.

**Table 3:** Comparison of average, maximum, and minimum planning times between clinical and auto‐planning strategies

N = 20				
Time Measured (N = 20)	Min	Average	St Dev	Max
Automated Plan (minutes:seconds)	04:48	05:31	00:42	06:21
Clinical Dosimetrist Time (minutes:seconds)	17:53	25:33	05:03	34:35
**Relative Planning Time (%)**	**27%**	**22%**	**14%**	**18%**
**Time Saving (minutes)**	**17**	**25**	**4**	**35**

## 4 DISCUSSION

APs were consistently better at reducing lung doses across all metrics. The significant improvements seen in auto‐planning is partially a function of the number of lung metrics assessed in the analysis. Of all heart metrics there were no significant variations between either planning technique, although APs tended to produce lower heart doses. It should be emphasized that the improvements are statistically significant but not necessarily clinically significant in all cases.

The trends in the statistics are supported by lower heart and lung doses on the DVH distributions in Figure 3. Another advantage of the automated planning implementation is an improved consistency in plan quality, with reduced first and third quartile ranges and standard deviations for the contralateral breast, ipsilateral lung, and heart distributions in comparison to clinical plans.

The contralateral breast mean dose metric was the only statistically significant improvement in the CPs for left‐sided patients. Conversely there appears to be a trend toward slightly higher contralateral breast doses in right‐sided CPs, although the difference is not statistically significant. Considering this effect is not mirrored in right‐sided cases and the slightly lower (although not significant) heart doses in the AP cases, this suggests a divergence of the relative importance given to heart and contralateral breast between human and software optimization, even in the presence of identical clinical goals.

A highly significant variation is noted in the 95% PTV coverage of right breast plans. This increase in coverage on the clinical plans is associated with an equivalent negative variation of mean dose (away from target dose) and maximum dose to the PTV, as well as a cost to the lung dose. Again, this highlights a difference between optimization strategies, with smaller ranges in variation between the AP metrics and a reduced emphasis on dose escalation at the cost of OAR metrics.

Compared to the clinical plans, automatically generated plans were consistently better performers against the three analyzed protocol dose regimes with 9%, 9%, and 5% more ideal plans by the RTOG, EviQ, and London Cancer protocols, respectively.

The automatically generated plans were thus equivalent, and in some cases superior, to clinical treatment plans. In most cases the superior AP plans were a result of a combination of very small changes in the open beam arrangements (gantry angle and reduced postedge margin) and slight under optimization in the clinical plans where optimization was ceased prior to the optimal solution as a result of favorable geometries that easily met clinical goals. Given that the hybrid breast technique is highly sensitive to beam angle selection, it is likely that the variation in selected gantry angles between the AP and manual plans also contributes to the variation in plan quality. Of the 28 plans where the gantry varied by more than 1 degree, automated plans selected a gantry angle that irradiated less tissue than the manual plans while achieving the planned clinical goals, suggesting that in some cases manual plans utilized suboptimal beam geometries. The overall mean gantry angle variation across the cohort reflects a strong similarity between user determined and AP selected gantry angles. The mean and variation across the cohort also correlates well with the results of Purdie et al.^4^ in which surface markers were implemented for angle detection.

In comparing the plan quality results against other studies, Purdie et al.^4^ showed similar equivalent plan quality variation between AP and manual plans. While their work did not result in any significant reduction in organ doses, a slightly different step and shoot IMRT method was incorporated in the study. Reductions in ipsilateral lung doses in automated plans are not unique to this study, with similar results across multiple planning systems by Mitchell et al,^10^ Wang, Sheng and Yoo,^6^ and Sheng et al.^12^ with techniques ranging from semiautomated planning to machine learning plan creation.

While the results suggest that automated planning can produce equivalent or even slightly improved plans to clinical dosimetrists, it is not the case that the automated plans necessarily produce the absolute most optimal plan, as demonstrated by the higher contralateral breast dose in left‐sided patients. A limitation of this study is that the metrics used do not allow for any particular dose distribution preferences of an oncologist not covered within the selected DVH metrics. Any future studies should incorporate a blind assessment by multiple oncologists of the CPs and APs. This was not possible in this paper due to availability of sufficient numbers of appropriate specialists at the time of research. Given the solutions adherence to clinical goal metrics it is likely that in some cases, APs may instead provide an initial near‐optimal plan that a dosimetrist or oncologist may wish to further optimize.

### 4.1 Deliverability

Automated and clinical plans show equivalent deliverability and QA results. Little can be drawn from the slight 1.1% improvement in average passing gamma points of the APs, with no statistical significance (*P* = 0.0714) owing to a small sample size. While a greater number of QA deliveries may result in statistical significance, as the primary concern of this paper is plan quality and efficiency gains, the current analysis is sufficient to show equivalence in deliverability and further QA measurements were not undertaken.

### 4.2 Planning time

The implementation of automated planning shows considerable advantages in both efficiency and workflow processes over manual clinical planning. The results in Table 3 show an average fourfold reduction in planning time as a result of the single session workflow change. It should be emphasized that the comparison here is of manual planning time free of interruptions. In clinical practice, it is likely that the actual planning times are significantly longer than those demonstrated in this paper due to possible interruptions and distractions, and as such efficiency gains from automated planning greater than reported.

The reported automated planning time presented in this study compares favorably with work by Sheng et al.^12^ (5 minutes) and Mitchell et al.^10^ (5 minutes 48 seconds) for plan creation. Work by Purdie et al.^4^ produced clinical plans in 6 minutes and 52 seconds inclusive of structure creation excluded in our study. All three studies above incorporated step and shoot IMRT whereas DMLC modulated planning was used in this work. The time cost related to modulated techniques is not investigated here but is likely to have an impact on results between the various papers.

This AP code can be run simultaneously on up to five patients on one terminal, providing further magnified efficiency improvements. With the help of automated contouring, together an oncologist and a dosimetrist can simultaneously run automated planning on multiple patients, review these completed plans, and make changes if necessary while waiting for the remaining plans to complete. In the initial development of the iterative optimization code, over 50 patients were able to be planned within 4 hours. This in particular opens up the possibility to a large change in the planning process of such patients. By incorporating high speed planning with dosimetrists into the oncologist target review workflow it is possible to eliminate the time cost of a separate contouring, planning and review session separated by hours or days depending on particular staff availability. There still remain potential issues with such a workflow with regards to patient simulation appointment scheduling—the advantage of simultaneous fast planning is negated if the contouring process for some patients are delayed for several hours after simulation to do so—and staff availability—oncologists often review contours late in the evening after patient consults are complete when dosimetrist are unavailable—however, the potential remains if logistical considerations can be addressed.

It should be noted that the current implementation of automated planning is not inclusive of auto‐contouring, which is accommodated by a separate script in the clinic. While as previously discussed, this results in a small time cost in comparison to some AP techniques that integrate auto‐contouring,^7^, ^8^ ensuring oncologists review contours prior to planning allows for flexibility of implementation with various techniques. By determining optimal tangent angles by adherence to approved oncologist contours, the code has the potential to support step and shoot IMRT, short‐arc breast VMAT, breast and node VMAT, and integrated boosts delivery techniques. The variability of nodal treatment extent poses a difficult problem for automated planning in the absence of volume review prior to plan creation.

Following the results of this research the described code has been implemented clinically at the authors’ site for both hybrid DMLC and step and shoot whole breast techniques.

## 5 CONCLUSION

The IronPython programming language has been used to develop an iterative optimization platform that automates the planning process of inversely planned radiotherapy. In application of the code on hybrid breast patients the automated plans have shown to be equivalent to clinically accepted plans across 100 retrospectively analyzed patients.

While plan quality and deliverability show little variance between the manual plans and the APs, vast efficiency improvements have been demonstrated for automated planning, with an average reduction in planning time of 78% from manual clinical planning as a result of the workflow change, allowing the completion of a plan in under 5 minutes.

By validating the automated planning code via comparison against accepted clinical plans, this paper has provided the grounding for the clinical implementation of automated hybrid IMRT breast planning in RayStation.

## AUTHOR CONTRIBUTIONS

Ben Archibald‐Heeren and Mikel Byrne were involved in the conception and design of the study. Data collection was performed by author Ben Archibald‐Heeren with analysis and interpretation conducted by authors Ben Archibald‐Heeren, Mikel Byrne, Yunfei Hu, Guilin Liu, Nick Collett, Meng Cai, Yang Wang. The article was drafted by author Ben Archibald‐Heeren with critical revision by authors Mikel Byrne, Yunfei Hu, Guilin Liu, Nick Collett, Meng Cai, Yang Wang.

## CONFLICT OF INTEREST

Ben Archibald‐Heeren, Mikel Byrne, Yunfei Hu, Guilin Liu, Meng Cai, and Yang Wang have no conflict of interest with regards to this work.

## References

[1] International Agency for Research on Cancer . World Cancer Report 2014. Lyon, France: World Health Organization; 2014.

[2] Ferlay J , Soerjomataram I , Ervik M . F. GLOBOCAN 2012 v1.1, Cancer Incidence and Mortality Worldwide: IARC CancerBase No. 11. Lyon, France: International Agency for Research on Cancer; 2014 http://globocan.iarc.fr/Pages/fact_sheets_cancer.aspx. Accessed February 20, 2018.

[3] Australian Institute of Health and Welfare . Radiotherapy in Australia 2015–16. Canberra: AIHW https://www.aihw.gov.au/getmedia/93ee58ce‐2ad1‐49fe‐86e3‐f552ff6ab54c/20730.pdf.aspx?inline=true. Accessed February 13, 2018.

[4] Purdie TG , Dinniwell RE , Letourneau D , Hill C , Sharpe MB . Automated planning of tangential breast intensity‐modulated radiotherapy using heuristic optimization. Int J Radiat Oncol. 2011;81(2):575‐583. 10.1016/j.ijrobp.2010.11.016. 21237584

[5] Purdie TG , Dinniwell RE , Fyles A , Sharpe MB . Automation and intensity modulated radiation therapy for individualized high‐quality tangent breast treatment plans. Int J Radiat Oncol. 2014;90(3):688‐695. 10.1016/j.ijrobp.2014.06.056. 25160607

[6] Wang W , Sheng Y , Yoo S , Blitzblau RC , Yin F‐F , Jackie Wu Q . Goal‐driven beam setting optimization for whole‐breast radiation therapy. Technol Cancer Res Treat. 2019;18:153303381985866. 10.1177/1533033819858661 PMC6598321 31242822

[7] Jiawei F , Jiazhou W , Zhang Z , Hu W . Iterative dataset optimization in automated planning: Implementation for breast and rectal cancer radiotherapy. Med Phys. 2017;44(6):2515‐2531. 10.1002/mp.12232. 28339103

[8] Fogliata A , Nicolini G , Bourgier C , et al. Performance of a knowledge‐based model for optimization of volumetric modulated arc therapy plans for single and bilateral breast irradiation. PLoS One. 2015;10(12):e0145137 10.1371/journal.pone.0145137. 26691687 PMC4686991

[9] van Duren‐Koopman MJ , Tol JP , Dahele M , et al. Personalized automated treatment planning for breast plus locoregional lymph nodes using hybrid rapidarc. Practical Radiation Oncology. 2018;8(5):332‐341. 10.1016/j.prro.2018.03.008. 29907505

[10] Mitchell RA , Wai P , Colgan R , Kirby AM , Donovan EM . Improving the efficiency of breast radiotherapy treatment planning using a semi‐automated approach. J Appl Clin Med Phys. 2016 10.1002/acm2.12006. PMC5689888 28291912

[11] Vanderstraeten B , Veldeman L , Dhondt R , Wagter CD , Lievens Y . Implementation and clinical evaluation of automated planning for IMRT treatment of breast cancer. Int J Radiat Oncol Biol Phys. 2015;93(3):E596 10.1016/j.ijrobp.2015.07.2068.

[12] Sheng Y , Li T , Yoo S , et al. Automatic planning of whole breast radiation therapy using machine learning models. Frontiers in Oncology. 2019;9: 10.3389/fonc.2019.00750 PMC6693433 31440474

[13] Byrne M , Hu Y , Archibald‐Heeren B . Evaluation of RayStation robust optimisation for superficial target coverage with setup variation in breast IMRT. Australas Phys Eng Sci Med. 2016;39(3):705‐716. 10.1007/s13246-016-0466-6. 27581727

[14] Mayo CS , Urie MM , Fitzgerald TJ . Hybrid IMRT plans—concurrently treating conventional and IMRT beams for improved breast irradiation and reduced planning time. Int J Radiat Oncol. 2005;61(3):922‐932. 10.1016/j.ijrobp.2004.10.033. 15708276

[15] Amoush A , Murray E , Yu JS , Xia P . Single‐isocenter hybrid IMRT plans versus two‐isocenter conventional plans and impact of intrafraction motion for the treatment of breast cancer with supraclavicular lymph nodes involvement. J Appl Clin Med Phys. 2015;16(4):31‐39. 10.1120/jacmp.v16i4.5188. 26218994 PMC5690023

[16] Shiau A‐C , Hsieh C‐H , Tien H‐J , et al. Left‐sided whole breast irradiation with hybrid‐IMRT and helical tomotherapy dosimetric comparison. Biomed Res Int. 2014;2014:1‐7. 10.1155/2014/741326 PMC4121994 25170514

[17] Thomas S , Hoole A . The effect of optimization on surface dose in intensity modulated radiotherapy (IMRT). Phys Med Biol. 2004;49:4919‐4928. 15584527 10.1088/0031-9155/49/21/005

[18] Offersen BV , Boersma LJ , Kirkove C , et al. ESTRO consensus guideline on target volume delineation for elective radiation therapy of early stage breast cancer. Radiother Oncol. 2015;114(1):3‐10. 10.1016/j.radonc.2014.11.030. 25630428

[19] Gentile MS , Usman AA , Neuschler EI , Sathiaseelan V , Hayes JP , Small W . Contouring guidelines for the axillary lymph nodes for the delivery of radiation therapy in breast cancer: evaluation of the RTOG Breast Cancer Atlas. Int J Radiat Oncol. 2015;93(2):257‐265. 10.1016/j.ijrobp.2015.07.002. PMC12422023 26383674

[20] Haciislamoglu E , Colak F , Canyilmaz E , et al. Dosimetric comparison of left‐sided whole‐breast irradiation with 3DCRT, forward‐planned IMRT, inverse‐planned IMRT, helical tomotherapy, and volumetric arc therapy. Phys Med. 2015;31(4):360‐367. 10.1016/j.ejmp.2015.02.005. 25733372

[21] Jeulink M , Dahele M , Meijnen P , Slotman BJ , Verbakel WFAR . Is there a preferred IMRT technique for left‐breast irradiation? J Appl Clin Med Phys. 2015;16(3):197‐205. 10.1120/jacmp.v16i3.5266. PMC5690145 26103488

[22] Vicini F , Freedman G , White J , et al. A Phase III trial of accelerated whole breast irradiation with hypofractionation plus concurrent boost versus standard whole breast irradiation plus sequential boost for early‐stage breast cancer. 2014 https://www.rtog.org/ClinicalTrials/ProtocolTable/StudyDetails.aspx?study=1005.

[23] Cancer Institute NSW . 1922‐Breast invasive cancer adjuvant EBRT conventional whole breast | eviQ. https://www.eviq.org.au/radiation‐oncology/breast/1922‐breast‐invasive‐cancer‐adjuvant‐ebrt‐conventi#34009. Published March 12, 2017. Accessed February 13, 2018.

[24] London Cancer . Guidelines for the treatment of breast cancer with radiotherapy. 2013 http://www.londoncancer.org/media/85204/london‐cancer‐breast‐radiotherapy‐guidelines‐2013‐v1.0.pdf. Accessed February 14, 2018.

[25] Wolstenholme V . Guidelines for the treatment of Breast cancer with radiotherapy. 2017 http://londoncancer.org/wp‐content/uploads/2017/10/London‐Cancer‐Breast‐Radiotherapy‐Guidelines‐2017‐version‐4.pdf. Accessed February 14, 2018.

